# Assessment of the Acceptability and Feasibility of Child Potties for Safe Child Feces Disposal in Rural Bangladesh

**DOI:** 10.4269/ajtmh.15-0932

**Published:** 2017-06-19

**Authors:** Faruqe Hussain, Stephen P. Luby, Leanne Unicomb, Elli Leontsini, Tania Naushin, Audrey J. Buckland, Peter J. Winch

**Affiliations:** 1 International Centre for Diarrhoeal Disease Research, Bangladesh (icddr,b), Dhaka, Bangladesh; 2 Stanford University, Stanford, California; 3 Department of International Health, Johns Hopkins Bloomberg School of Public Health, Baltimore, Maryland

## Abstract

Indiscriminate defecation among young children and the unsafe disposal of their feces increases fecal contamination in the household environment and the risk of diarrheal disease transmission. Improved sanitary technology for children too young to use a latrine may facilitate safe feces disposal and reduce fecal contamination in the household environment. We assessed the acceptability and feasibility of child potties in rural Bangladesh in 2010. Our team introduced child potties into 26 households for 30 days, and conducted semistructured interviews, group discussions, and observations to assess the acceptability and feasibility of their use for parents and children. Residents of this rural Bangladeshi community accepted the child potties and caregivers found them to be a feasible means of managing child feces. The color, shape, design, and size of the potty influenced its acceptability and use. These residents reported that regular use of the potty improved the household’s physical environment and caregiver and child personal hygiene. Regular potty use also reduced caregivers’ work load by making feces collection and disposal easier. Primary caregivers viewed 4–6 months as the appropriate age to initiate potty training. Sanitation interventions should integrate and emphasize potties for children’s feces management to reduce household environmental contamination.

## INTRODUCTION

Globally, diarrheal illness is a leading cause of childhood morbidity and mortality.^1^^,^^2^ Open defecation and unsafe disposal of feces increases the risk of disease transmission.^3^^–^^6^ An improved sanitary environment may reduce childhood diarrheal incidence substantially by interrupting fecal–oral transmission.^7^^–^^10^ Safe disposal of children’s feces was associated with reduction in helminth infestation in children under 2 years of age in rural Bangladesh.^11^

The safe disposal of child feces is an important component of child health because feces present in a child’s environment can expose them to diarrheal pathogens and parasites.^12^ Young children in rural Bangladesh do not usually wear diapers,^13^ and few use potties (a bowl/pot/container used by small children as a toilet). Thus, open defecation is the norm among young children in Bangladesh, as also reported in the Philippines,^7^ Indonesia,^14^ Sri Lanka,^15^ Burkina Faso,^16^ and Peru.^17^

Bangladeshi infants are commonly wrapped in a thin, home-made wrap (*katha*) made of several layers of used traditional cloth.^18^ Until 6 months of age, babies defecate in their mothers’ lap, on a bed, or in a *katha* that captures the feces. These in turn are washed in the nearest water sources such as a pond, canal, or river, and sometimes on a tube-well platform.^19^ In a study conducted by our research team in rural Bangladesh, the use of child potties was limited.^19^ Caregivers often collected and disposed of their children’s feces in unhygienic ways such as picking up the feces with leaves, straw, and paper, or scooping up the feces with a small hoe (*seni*) commonly used for weeding.^19^

Adult latrines are not designed or suitable for very young children. Less than 1% of mothers in a study in Burkina Faso reported that their children 36 months or younger used adult latrines.^20^ Peruvian mothers reported that their young children were unable to use adult latrines in a sanitary way.^21^ Similarly, in Bangladesh, many children start using a latrine when they are 3–4 years of age.^19^ Parents fear that without supervision and assistance, young children may fall into the latrine hole or injure themselves. However, young children rarely use potties in most low-income countries, including in south Asia because potty training is considered difficult and time consuming.^17^^,^^22^

Although various potty models are available in local markets at a range of prices, typically only wealthy parents use a potty for their children. Potty training for children in rural Bangladesh is very limited,^19^ and is a relatively new behavior. Rural parents are not aware of the advantages of using a potty or may not know how to potty train their children. Changing child defecation practices is difficult once they form the habit of open defecation.

A child friendly and socially acceptable method for feces disposal would encourage caregivers to adopt consistent hygienic disposal of child feces. We report on formative research that used a small-scale household trial of improved practices (TIP) to assess the feasibility and acceptability of child potties among caregivers of children < 3 years of age in rural Bangladesh.

## METHODOLOGY

### Study site and population.

We conducted this formative research study in a rural subdistrict of Kishoreganj, a northern district of Bangladesh, during June and July 2010. Study participants included mothers of children 7–36 months of age as primary caregivers and fathers and grandmothers as secondary caregivers. We considered children 7–36 months to be within the optimum age for initiating potty training.

### Study design.

Formative research is a systematic approach to obtaining data from community members that can be used for tailoring behavioral components of an intervention to a specific local context.^23^ TIPs is a participatory formative research methodology used in public health intervention studies to test the acceptability and feasibility of new practices within a community or household by a small group of participants selected from the larger population.^24^ Participants are considered experts on the behavior of interest. An acceptable behavior is one in which participants are willing to adopt and practice, that is feasible, practical, beneficial, and can be adjusted through negotiation.^24^ By providing feedback, the participants teach the researchers what is acceptable and feasible within the physical and social environment in which they live.^25^ TIPs have been used to develop diverse interventions including nutrition, diarrheal illness prevention, hygiene, and malaria prevention.^24^^,^^26^^–^^30^

We assessed the acceptability and feasibility of potty training, potty use, and maintenance using the TIPs methodology. This formative research was then used in the selection of an enabling technology, the child potty, and the accompanying behavior change strategy for a larger trial assessing the effects of different combinations of water, sanitation, hygiene, and nutrition interventions in rural Bangladeshi households on child health and nutrition outcomes.^31^

### Sampling.

The study was conducted in two rural villages from the Katiadi subdistrict of the Kishoreganj District in Bangladesh. Households were eligible for inclusion if they lived in an easily accessible compound and had a child 7–36 months of age. Field workers went to each village and sequentially visited all of the households within a cluster (groups of homes in a village). The field team approached the adult members and explained the research objectives, asked if there were any children of eligible age living in the household and enrolled 10–15 households in each village for a total of 26 households. Two households each had two eligible children.

### Introduction of the potties and household visits.

We conducted baseline semistructured interviews with child caregivers from the 26 households to collect basic demographic information and to explore current sanitation facilities of the households, existing child defecation practices, presence of child feces, feces disposal practices, and access to potties.

The research team selected three locally available child potty models. Differentiating features included shapes, a removable head, removable pot, and lid (Table 1). Field workers met with caregivers and introduced the potty models using pictures. Each caregiver selected a model and received their choice of potty for free. During this home visit, field workers explained to caregivers that young children may resist sitting on the potty or may not be interested in defecating in the potty. Our team advised caregivers not to force children to sit on the potty if they refused initially, but rather to increase familiarity, and encourage them to play with it.

**Table 1:** Description of potty models

Photo	Name	Number provided*	Description
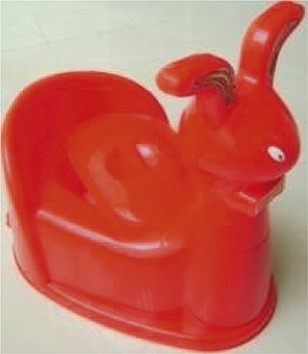	Rabbit	10	Plastic body with removable rabbit head
			Ears for child to grasp
			Smooth seat
			Removable pot under seat
			Lid to cover potty hole
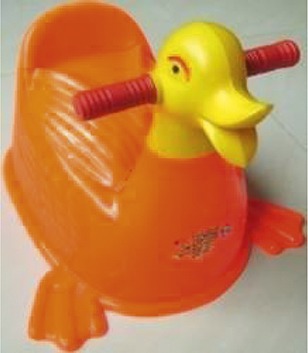	Duck	7	Plastic body with duck head
			Two handles for child to grasp
			Two feet for stability
			No removable pot
			Lid to cover potty hole
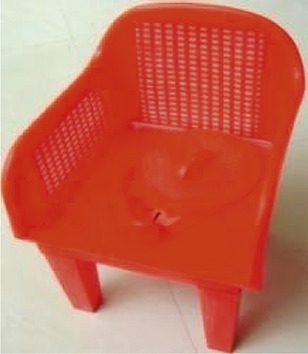	Chair	11	Plastic body shaped like a chair
			Removable pot under seat
			Lid to cover potty hole

* Two household had two children under 3 years of age.

The day after distributing the potties, caregivers were invited to a common space to share their experiences introducing the potty and initial problems confronted. During subsequent follow-up visits, interviewers conducted semistructured interviews to explore several topics including: potty familiarization, introduction of potty training, problems children encountered while defecating in the potty, potty cleaning and maintenance, benefits of and barriers to potty use, and location of feces disposal. The team visited each household five times in 30 days for data collection (Table 2). On the third visit, field workers observed the children’s first defecation event in the morning. Interviewers took notes during all follow-up visits using a semistructured instrument.

**Table 2:** Data collection methods and schedule

Visit no.	Timing	Data collected	Methods
1	Day 1	Initial feedback on hardware	Group discussion
2	Day 3	Hardware problems	Individual interviews
		What is liked and disliked about potties	Group discussion
3	Day 7	Potty introduction process	Informal conversation
		Potty use by caregiver and child	Observation of child’s first defecation event
		Feces disposal practices	
		Hardware maintenance	
4	Day 14	Identify barriers to use	Group discussion
		Identify challenges and benefits	Individual interviews
5	Day 30	Newly emerged problems	Group discussion
		Recommendations	

### Data analysis.

Our team sorted the responses into subthemes including potty benefits, introduction methods, potty use and maintenance, suggestions for use, feces disposal practices and disposal site, potty training difficulties, and problems encountered. We applied the integrated behavioral model for water, sanitation and hygiene (IBM-WASH) to summarize the data.^32^ We organized and analyzed data from interviews following the three main dimensions of the model. The study team identified contextual, psychosocial, and technology factors that influenced the acceptability and feasibility of potty training, potty use, and maintenance at the community, interpersonal/household, individual, and habitual levels as per the model.

### Protection of study participants.

Our research team explained the objectives of the study to the participants and obtained their informed consent. The participants signed the consent form and those who were not able to sign provided their thumbprint. The study was conducted under the WASH Benefits pilot study protocol (no. 9053) that was reviewed and approved by the Committee for the Protection of Human Subjects (Ethical Review Committee and Research Review Committee), by the International Centre for Diarrhoeal Disease Research, Bangladesh.

## RESULTS

### Reactions to introduction of the potties.

Twenty-four caregivers (92%) were homemakers and 50% had completed primary education. We included 28 children in the study: 21% were under 12 months, 43% were 13–24 months, and 36% were 25–36 months of age. Most (96%) households had access to a latrine (individual 59% and shared 37%).

Before potty distribution, it was common for caregivers to leave feces where the child had defecated, resulting in visible child feces in the courtyard (Table 3). Many caregivers acknowledged that feces disposal was considered “dirty” in the community, and they felt uncomfortable using leaves, straw, or paper to dispose of feces. One mother said,

I used to throw the feces into the bush or field around the homestead. No matter whose feces it is, it stinks and is disgusting. Collecting feces with leaves or straw does not remove the feces completely, I have to rub the spot with my feet putting a little water to eliminate the feces stain.

After potty distribution, caregivers reported that their neighbors appreciated their potty use to manage their children’s feces. Several neighbors expressed a desire to use potties with their own children. Using a potty for child defecation portrayed the image of a “clean mother” to neighbors. One mother explained,

No one can criticize me as a ‘dirty mother’ because I am using a potty to manage feces disposal. I am proud of using a potty!

**Table 3:** Caregivers’ report of child defecation and feces management practices before and after the trial

Behaviors/practices	Baseline visit (%)	After the intervention (%)
Child defecation place (28 children)		
Within the courtyard ground	24 (86)	4 (14)
Outside the homestead	3 (11)	0 (0)
In potty	1 (4)	24 (86)
Feces collection method (26 households)		
By bare hand using leaves/straws/papers	24 (92)	0 (0)
By hoe/scoop	2 (8)	3 (12)
By potty (stopped use)	0 (0)	23 (88)
Feces disposal site (26 households)		
Thrown in bush	12 (46)	0 (0)
In waste pond	8 (31)	4 (15)
In garbage pit	5 (19)	0 (0)
In latrine	1 (4)	22 (85)
Buried	0 (0)	0 (0)

### Household roles and potty use.

The division of labor within the household was the main factor identified that affected the feasibility of regular use of child potties. The introduction of a potty along with potty training required assuming responsibility for additional tasks: giving the potty to a child for play; holding the younger child over it; encouraging the child to stay on and defecate; disposing feces into a latrine; and washing, drying, and storing the potty for the next use (Table 4). Older siblings observed caregivers and learned how young children should use the potty, how to pull out the removable pot and how to put it back properly. When a mother was busy with her regular household chores or was absent for a while, older siblings cleaned the potty and set it out to dry. One mother described this process,

My elder daughter helped the baby to defecate in the potty. When the younger baby completed defecation she washed the potty and kept it inside the room under the bed.

Caregivers could cover the potty with a lid to contain the odor and prevent the attraction of flies if they were not ready to immediately dispose of the feces (Table 5). Mothers reported covering the potty when their child defecated at night or when they were sick or busy with other household chores and were unable to immediately clean the potty. Caregivers cleaned the potty in the morning if the child had defecated at night, or when they felt better, or had available time. One mother explained this,

Once I felt sick and my child defecated in the potty. I covered the potty and cleaned it when I felt better and it was really convenient.

**Table 4:** Observation of potty use by children (*N* = 28)

Behaviors	*n* (%)
Defecated in potty	16 (57)
Feces visible in pot	9 (32)
Signs of immediate feces cleaning	7 (25)
Potty cleaning place/location	
In latrine	10 (35)
Under hand pump/tube well	4 (14)
In the courtyard	2 (7)
Accompanied child during defecation	
Mother	6 (21)
Grandmother	2 (7)
Sister/aunt	3 (11)
Child defecated alone	4 (14)

**Table 5:** Benefits of potties perceived by the caregivers

Benefits mentioned (multiple response)	*n* (%)
Reduced work load	
Saves time by collecting feces	24 (92)
No need to search for leaves/straws/papers	18 (69)
Child can defecate alone	14 (54)
Can empty feces later (cover the potty)	25 (96)
Children play with potty	15 (58)
Improved personal hygiene	
Feces do not come in contact with hand	16 (62)
Children do not touch the feces	18 (69)
Children do not play with feces/get dirty	20 (77)
Improved household environment	
No bad smell	25 (96)
Courtyard remains clean	20 (77)
Barriers mentioned (multiple responses)	
Child resisted sitting	4 (15)
Feces stuck to the pot	2 (8)
Attention and time is required to dry potty	2 (8)
Potty size is not conducive to child’s size	3 (12)

In addition to caregivers and older siblings, grandmothers and aunts also helped children to use the potty, emptied feces, and cleaned the potty. They helped young children sit on the potty, kept them busy by giving toys or objects, and guided them to hold on the handles for stability. Fathers of the children appreciated the potty training but did not actively participate in the training process.

### Challenges to potty use.

When caregivers first tried to make children sit on the potty, many of the children irrespective of age were intimidated (Table 6). Over the study period, children became familiar with the potty. However, there were notable differences in the acceptability of potties between younger and older children, indicating that the developmental stage of a child affects potty use. Young children, under 1 year, were initially frightened to sit on the animal shaped potties, whereas older children in general treated the potty as a toy. Caregivers had to hold children under 1 year of age on the potty or persuade them to stay on it until defecation was completed. Caregivers considered this effort to be too time consuming. Additionally, some of these young children were too small to easily sit on the potties.

**Table 6:** Caregivers’ report of problems encountered by children during potty training by their age

Problems	Age		
	< 12 months	13–24 months	24+ months
Child fell off of the potty	3		1
Child got scabies on buttocks	2		
Child is scared to sit	1	2	1
Potty is large in size	1		
Potty is small in size		2	1
Child would not defecate		4	
Painful for child to sit on	5		1

Older children, 13–24 months of age, would usually sit on the potty, but some resisted defecating into it. Caregivers explained that these children had already developed the habit of defecating indiscriminately in the open. Children over 2 years of age were accustomed to defecating indiscriminately and most refused to sit on the potty to defecate. Some children over 2 years of age, however, liked the potties (3/8), and retrieved and used them without prompting. One mother commented,

The child can alone bring the potty and defecate in it. I don’t need to help him. So I can do my household work without interruption.

Household access to latrines influenced the feasibility of using potties. Caregivers with access to their own improved or unimproved latrines used these for emptying the potty. Those who did not own a latrine but shared with other households generally disposed the child feces either into the latrine or a designated pit.

### Potties as reminders or cues to action.

The potty presence served as a cue to action for use among children. Many caregivers kept the potty in a visible and easily accessible location such as under the table or the bed. A second cue to action was the child’s persistent interest to play with the potty. These cues to action supported potty training habit development. All the household members considered using child potties a good practice that would eventually encourage “good habits” in their children. One of the caregivers expressed her aspiration saying,

Gradually my child is becoming habituated with potty use. Today I didn’t force her but she willingly sat on it (potty) and completed defecation. When she grows up she will never defecate in the open.

We cannot definitively say that children developed long-term potty training habits due to the short duration of this study. Some children only urinated in or played with the potty but did not defecate, which indicated partial use. Caregivers were encouraged to promote urination in the potty as a first step to familiarization. Child age was reported as a determinant for habit formation. The mother of a 15-month-old child said,

I think if you would have given the potty when the child was less than 6 months old he would be familiar very quickly. Now he is more than 1 year old, so we are facing problems to make him use it on a regular basis.

### Influence of potty design.

The design and availability of child potties influenced the acceptability and feasibility of its use. Several of the potty models available in the local market were not favored by the caregivers because they were very light or simple in design without a removable pot, lid, or handles. However, several caregivers mentioned that they might not be able to afford such a high-quality potty as the model provided. Caregivers noted the lid of a potty as a benefit of the design because they could cover the potty when they were busy and clean it at a more convenient time. The sitting surface was an important factor for acceptability among children. The chair potty was less popular because it did not have a smooth seat, and so risked scratching the child’s buttocks. During the initial household visits, only one household was found with a potty, but the mother reported that her child had fallen off the potty once and thus she was not interested in using it. This household was provided with a rabbit-shaped potty by the study and the child started to use it. The caregiver described that her daughter liked the sturdy potty because she could hold on to it and play with it. Through the introduction of a more attractive and sturdy potty with handles and a smooth seat, the child was successfully reintroduced to potty training.

Designs with a removable pot were favored because this feature made feces collection and disposal more convenient. Two of the potty models introduced to the community had a removable pot, but the rabbit-shaped model with the removable head, pot, and lid was the most accepted and feasible. The removable pot could be pulled out and carried to the latrine for disposal and washing without distracting children’s play.

Caregivers demonstrated initiative by putting some water into the potty before their child defecated which made it easier to dispose of the feces. By the end of the study, caregivers could carry the removable pot to the latrine, appropriately dispose feces into the latrine, clean and dry the potty, and store it in an easily accessible location such as on the table or under the bed for later use.

Caregivers perceived that regular potty use improved the personal and home environment. Potty use separated feces from the surrounding environment in two ways: it prevented chickens from spreading feces and children from touching the feces. Previous feces disposal practices contaminated the environment if caregivers threw feces into the bush or ditches from where poultry could bring it back to the courtyard. Mothers had to clean the feces immediately after a child defecated on the ground, otherwise children could touch the feces, soil their bodies with it, or even put it into their mouths. One mother said,

Earlier I threw the feces into the bush or in the backyard. The chickens and ducks usually scavenged in those places and roamed making the courtyard dirty. But now I dispose the feces into the latrine. Now we stay cleaner and our courtyard is free from our children’s feces.

Potties made feces collection easier because the caregiver could collect all the feces in one location. One caregiver stated,

It was time consuming to remove and clean feces from different spots (when the child would defecate indiscriminately without the potty). But potty use saved my time and I can use the time for another purpose (work).

## DISCUSSION

In our rural Bangladesh study site, caregivers accepted potty training for young children and we found locally available potty models that were feasible to use. Defecating in a potty was a new practice for young children and the disposal of feces from a potty was a new behavior for their caregivers. We identified factors that influenced child defecation behaviors and the adoption of safe disposal practices for child feces within the contextual, psychosocial, and technological dimensions of IBM-WASH.^32^ We summarize the results according to these dimensions below.

### Contextual factors.

The contextual dimension of the IBM-WASH model refers to the physical environment, roles, and responsibilities of household members. Although mothers typically are responsible for child defecation and feces management activities in rural Bangladesh, our study showed a shift in this division of labor. Mothers received support from their eldest daughter along with grandparents and aunts when potties were used for child defecation. Support from household members when the mother is busy, sick, or away from home helps to ensure consistent potty use.

Various individual factors influence toilet training initiation for young children (< 3 years) including age and developmental stage of the child. At an early age, children are inclined to discover and enhance their physical abilities.^33^ By 6 or 7 months, most children are able to sit,^34^^,^^35^ which is part of the assessment of a child’s readiness for toilet training.^33^^,^^36^ Initiating toilet training during the first 6 months has the possibility of earlier completion,^37^ as suggested by participants in this study. Caregivers in Burkina Faso introduced potties to their children at under 6 months of age.^20^ Introducing potty training at an early age is advantageous for caregivers who use diapers since potty use can save time and money.^17^^,^^38^^,^^39^ Older children (around 24 months) in our study resisted sitting still and were reluctant to defecate in the potty.

#### Psychosocial factors.

The psychosocial dimension in the IBM-WASH model refers to the social and behavioral factors that influence behavior change and the adoption of an enabling technology.^32^ Descriptive norms refer to what people perceive others in the community to be doing.^40^ The descriptive norms in our study area were open defecation by young children, with caregivers scooping up feces with straw, leaves, or a small hoe. Participants did not like the existing disposal methods but had continued with them, suggesting lack of alternatives.

Self-efficacy and aspirations influence WASH practices.^41^^–^^43^ This intervention built the self-efficacy of caregivers to potty train their children and caregivers expressed aspirations for their children to develop “good habits” and to not defecate outside when they grow up.

#### Technology factors.

The final dimension in the IBM-WASH model is technology, which refers to the physical qualities of an enabling technology which influence its adoption.^32^ Design, quality, and size influence the acceptabilty and feasibility of child potties. Our findings were consistent with a study conducted in a Peruvian shanty town which identified that falling from a potty often resulted in potty training failure.^17^ The study was able to identify a locally available stable potty design that did not result in the child’s falling. Furthermore, the removable pot, and lid greatly facilitated ease and convenience of use by the caregiver.

Using a potty saved time by reducing caregivers’ work load. Our findings are consistent with the experiences and perceived benefits reported by Peruvian mothers. In addition, our caregivers’ innovation of putting some water in the pot before a child defecated eased the emptying which was similar to the technique introduced by some Peruvian mothers to make the feces disposal easier.^17^ Potty use had an unintentional advantage in that children spent time playing with the potty which gave mothers the freedom to do household chores.

Although our study was not designed to document habit formation, based on our findings, the provision of a potty provided a favorable, stable environment for habit formation among children and parents. The ease of potty use by children and maintenance by parents enabled the easy repetition of the associated behaviors, and the physical presence of the potty served as a cue to action for the children.

Throwing children’s feces into a bush or nearby field is easier than disposing of them in a latrine or specific disposal site. Contamination of the courtyard environment with children’s feces could be reduced through consistent potty use combined with feces disposal into a latrine. Helminth infestation may not decrease among children if caregivers do not remove child feces safely from the household environment.^11^

### Study limitations.

A limitation of this formative research study was that we handed out the potties free of charge. Affordability factors for potties need to be researched before they can be promoted programmatically. Another limitation was the short follow-up period of 30 days; therefore, we could not investigate the completion of potty training. During this period, we were able to capture caregivers’ feedback on different potty models, problems encountered by children during potty training, and influencing multilevel determinants.

## CONCLUSIONS

Effective sanitation programs need behavior change interventions accompanied by an appropriate enabling technology. Ownership of a household latrine is a contributing factor to enable safe child feces disposal practices,^12^ but is not sufficient to ensure hygienic feces disposal practices. Four behaviors should be promoted in a child potty behavior change intervention for safe disposal of children’s feces: 1) acquisition of a potty, 2) potty training, 3) regular emptying of the potty into a latrine or safe burial of feces, and 4) cleaning and maintenance for continued use.

The design and characteristics are key considerations for the acceptability of using child potties in rural Bangladesh. There is no specific recommended age to initiate toilet training, and children under 5 years of age rarely use latrines in rural settings. A potty can be used as early as children are interested and feel comfortable, in our context, as early as 6 months of age. After children abandon the potty, caregivers should be guided to train their children to use the adult latrines. Grandmothers, aunts, or older siblings can be the most appropriate secondary caregivers to support potty training, emptying feces into a latrine, and guiding children to use the adult latrine to ensure that open defecation does not occur during the transition period.

In Bangladesh, almost 99% of the population has access to a latrine or other form of sanitation, but only 48% have access to an improved, not shared, sanitation facility.^44^ Improvement in sanitary facilities and the safe disposal of child feces together would further reduce the exposure to fecal pathogens in the environment.
